# Gait-Specific Optimization of Composite Footwear Midsole Systems, Facilitated through Dynamic Finite Element Modelling

**DOI:** 10.1155/2018/6520314

**Published:** 2018-12-23

**Authors:** Dimitris Drougkas, Evagelos Karatsis, Maria Papagiannaki, Serafeim Chatzimoisiadis, Fotini Arabatzi, Stergios Maropoulos, Alexander Tsouknidas

**Affiliations:** ^1^BETA CAE Systems S.A., 54005 Thessaloniki, Greece; ^2^Department of Physical Education and Sport Science, Aristotle University of Thessaloniki, Ag. Ioannis, 62122 Serres, Greece; ^3^Department of Mechanical Engineering & Industrial Design, Technical University of Western Macedonia, Koila, 50100 Kozani, Greece

## Abstract

**Objective:**

During the last century, running shoes have been subject to drastic changes with incremental however improvements as to injury prevention. This may be, among others, due to the limited insight that experimental methodologies can provide on their 3D in situ response. The objective of this study was to demonstrate the effectiveness of finite element (FE) modelling techniques, in optimizing a midsole system as to the provided cushioning capacity.

**Methods:**

A commercial running shoe was scanned by means of micro computed tomography and its gel-based midsole, reverse-engineered to a 200 *μ*m accuracy. The resulting 3D model was subjected to biorealistic loading and boundary conditions, in terms of time-varying plantar pressure distribution and shoe-ground contact constraints. The mesh grid of the FE model was verified as to its conceptual soundness and validated against velocity-driven impact tests. Nonlinear material properties were assigned to all entities and the model subjected to a dynamic FE analysis. An optimization function (based on energy absorption criteria) was employed to determine the optimum gel volume and position, as to accommodate sequential cushioning in the rear-, mid-, and forefoot, of runner during stance phase.

**Results:**

The in situ developing stress fields suggest that the shock dissipating properties of the midsole could be significantly improved. Altering the position of the gel pads and varying their volume led to different midsole responses that could be tuned more efficiently to the specific strike and pronation pattern.

**Conclusions:**

The results suggest that midsole design can be significantly improved through biorealistic FE modelling, thus providing a new platform for the conceptual redesign and/or optimization of modern footwear.

## 1. Introduction

Historians often portray the 1970s as a “pivot of change” for the industrial world [1], both economically and culturally. The latter part of that decade also redefined what we perceive today as recreational sport, giving birth to a fitness evolution that was bound to change the lives of millions around the globe [2]. The so-called “running boom” drove masses into activities ranging from jogging to competitive road running, gaining momentum ever since, while simultaneously precipitating the development of the first technical running shoe [3]. As a result of this constantly growing movement, the athletic apparel industry has been subject of an unprecedented growth [4], which allowed technical footwear design to arguably mature into one of the most advanced branches of ergonomics.

Despite this and the fact that running biomechanics have been exhaustively studied over the past years, footwear improvements are often characterized as incremental in terms of injury prevention [5, 6]. Although the use of composite materials and complex midsole design has taken the footwear industry by storm, experimental methodologies struggle to provide comprehensive insight into the 3D in situ response of the structure. This 3D aspect is however fundamental to a holistic midsole design, as impact dynamics are inherently not only a time- but also a space-varying phenomenon [7]. As a result, most newly introduced technologies fail to live up to their full potential [8].

Over the past decade, finite element (FE) modelling has emerged as a valid alternative to in vitro [9] and in vivo testing [10], gaining wide acceptance within the biomechanics community. Despite this, the majority of studies employing FE methods in footwear modeling are either acknowledging only static loading conditions [11] or subjecting to geometry-based simplifications such as axisymmetry [12] and are thus not able to evaluate the system's in situ dynamic response.

Other limitations of current literature are associated to a focus on specific anatomical sites (e.g., the heal pad [13]). Although a recent study [14] has pointed towards the capability of simplified models to predict plantar pressure for patient specific considerations, such models are likely not acceptable while seeking redefined insight into footwear biomechanics, as running shoes are subject to swiftly altering dynamic loads [15] and contact conditions at the shoe-ground interface [16].

The hypothesis of this investigation is that advanced FE modelling, coupled with sensory feedback devices (in situ plantar pressure measurements), could facilitate the consideration of these finite boundaries, while being able to provide comprehensive feedback, of the interactive effects of midsole materials and running dynamics, on the structure's capacity to mitigate the occurring impact. If proven true, this could be a key-enabling approach to fully utilize on the properties of advanced materials, in developing a new generation of functionalized footwear systems.

The purpose of this study, towards this end, was to evaluate the effectiveness of FE modelling techniques, in optimizing a midsole system as to its shock-mitigating capacity. The developed 3D model improved current literature both in terms of biorealistic/dynamic loading scenarios and geometry accuracy of the midsole system.

## 2. Materials and Methods

**Figure 1:**
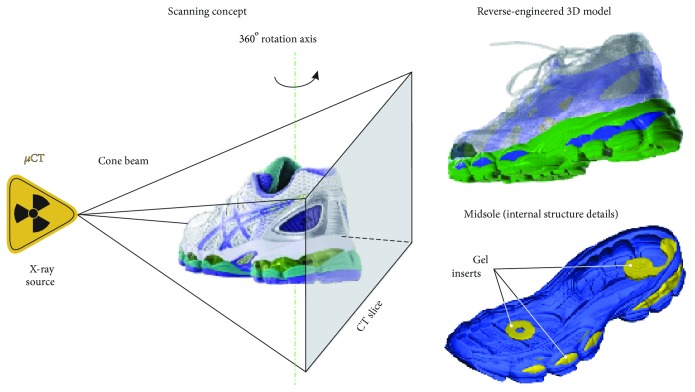
Reverse engineering concept and resulting 3D model.

A commercial running shoe was scanned by means of a micro computed tomography device (Werth TomoScope® HV Compact-225 3D CNC) and its gel-based midsole, reverse-engineered to a 200 *μ*m accuracy. The resulting 3D model (see Figure 1) consisted of a polymer foam and four gel inserts, a 12.7 ml one placed under the heel and three further ones, structured in the forefoot region of the shoe.

### 2.1. Loads and Boundary Conditions

**Figure 2:**
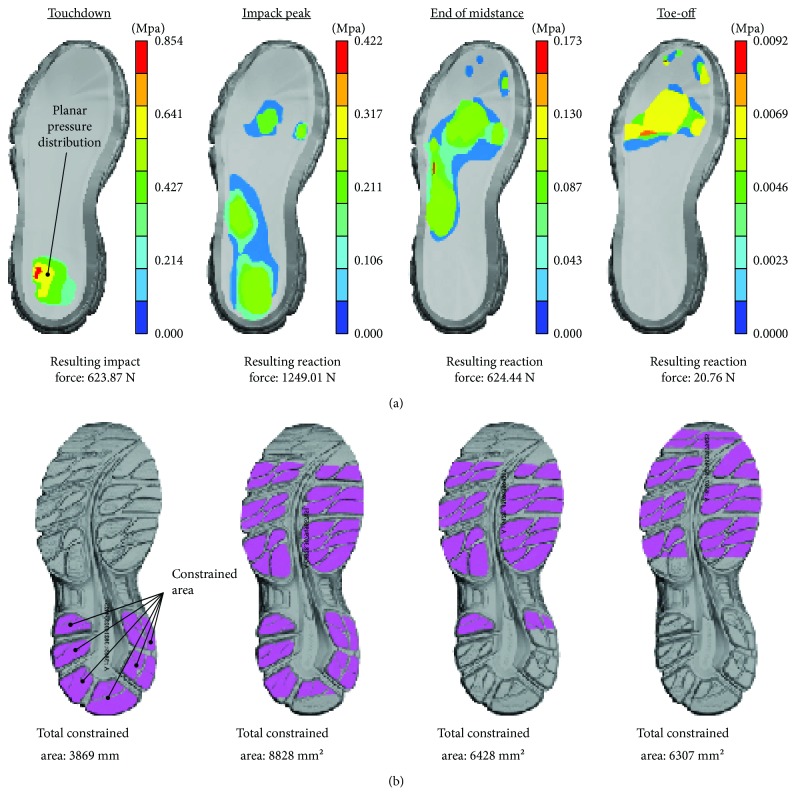
(a) Indicative load steps and (b) subshoe boundary condition considered during the analysis.

The plantar pressure distribution, occurring during running, was determined by a Footscanner insole 2.39 system (Niceville, FL, 32578, USA), whereas the time-dependent shoe-ground contact was extracted from high-speed camera measurements (MotionBLITZ EoSens® mini). The plantar pressure was recorded for a 51 kg female endurance athlete and the results filtered and statistically normalized. Both load and boundary conditions were applied under dynamic conditions to the superior and inferior surface of the cushioning system. Four characteristic phases of the modelled stance cycle (touchdown, impact peak, end of mid-stance, and toe-off) are illustrated in Figure 2.

These loading scenarios coincide well with existing literature, applying plantar pressure distributions for FE modelling approaches, even though most of these focus on walking scenarios with specialized footwear, e.g., high heels [17].

### 2.2. Material Properties

The midsole matrix (base material) was considered as a dual-density compression molded ethylene-vinyl acetate (CMEVA) foam, with a 0.95 g/cm^3^ density and 0.32 moles of elastic active chains/liter of cross-linked network [18]. Since the midsole is accepted to function within its elastic range, the transient viscoelastic modulus *E*_CMEVA_(*t*) was represented through the sum of exponents [19] of equation (1).

(1) $$\begin{array}{c} E_{CMEVA}\left(t\right)=\overset{3}{\underset{i=1}{\sum }}E_{i}e^{-t/\tau_{i}}+E_{\infty }, \end{array}$$

where the elastic constants and respective time relaxation values are *E*_1_ = 160 kPa at *τ*_1_ = 21.6 min, *E*_2_ = 130 kPa at *τ*_2_ = 86.4 min, and *E*_3_ = 55 kPa at *τ*_3_=507.6 min, whereas the long-term elastic modulus (*E*_∞_) was fixed at 2650 kPa.

The complex nonlinear stress-strain response of the gel pads within the CMEVA was represented as a hyperelastic material. The strain energy density of the gel was approximated through an Ogden material model formulation with the following material constants: *N* = 2, shear moduli of *μ*_1_ = 1000 kPa and *μ*_2_ = 50 kPa, and Poisson's ratios of *ν*_1_ = 0 and *ν*_2_ = 0.4 and *a*_1_ = 10 and *a*_2_ = −4.

### 2.3. FE Model

The mesh grid was generated in ANSA (BETA CAE Systems), facilitating a targeted meshing and consideration of fully conforming interfaces. This approach ensured a smooth stress transfer in between model entities, while maintaining a sufficient degree of computational efficiency.

The mesh grid of the FE model was verified, through convergence studies, as to ensure its conceptual soundness. Identifying the optimum mesh density in terms of processing time vs. results accuracy resulted in a mesh independent grid while maintaining vital geometric characteristics (feature lines) throughout the model. This was achieved by gradually decreasing the element size in all individual model entities up to a point where results were distorted by less than 0.2% [20]. Some of the element quality criteria are summarized below.

**Figure 3:**
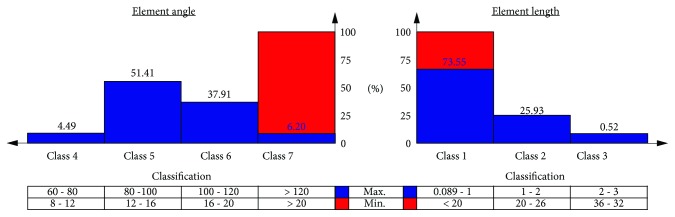
Mesh-related quality criteria.

The final model consisted of five entities, one CMEVA matrix corresponding to the 89.22% of the midsole volume and 4 gel volumes (modelled as separate entities to allow volume-specific optimization). The entire model consisted of tetrahedral elements, with skewness values ranging from 0.10 to 0.99 (class 2), where 1 corresponds to a theoretically “perfect” tetrahedral element. About 92% of the elements exhibited an aspect ratio between 1 and 2 (class 2), whereas the remaining 8% had a max aspect ratio of about 4 (class 3). Further element quality criteria are summarized in Figure 3.

The model was validated against velocity-driven impact tests, in accordance to ASTM F1976-06, conducted on an automated device (INSTRON CEAST 9350) [21]. A trend validation process was chosen for the model; focusing on these terms was not on the accurate approximation of the experimental data, but on the model's ability to simulate “trends.” For example, a variation in the experimental setup, leading to an increase of the midsole's cushioning capacity, does result in the calculation of a similar percentile increase (FE model).

### 2.4. FE-Driven Midsole Optimization

An optimization function was introduced to the model as to evaluate the effectiveness of the different gel pads' positioning and shape. The search for an optimal solution (providing high-energy absorption and support) was driven by optimization algorithms provided by modeFRONTIER (by ESTECO SpA, Italy).

The provided support was considered as to lead the optimization away from midsole structures with significantly increased gel volumes, which would arguably provide exceptional cushioning at the cost of low support. Shoe support was considered in terms of the angulation of the midsole's transverse plane. This “balance” was determined by measuring the axial displacement of contralateral nodes along the shoe's anteroposterior axis and set as a constraint during the optimization of the midsole's absorption capacity.

**Figure 4:**
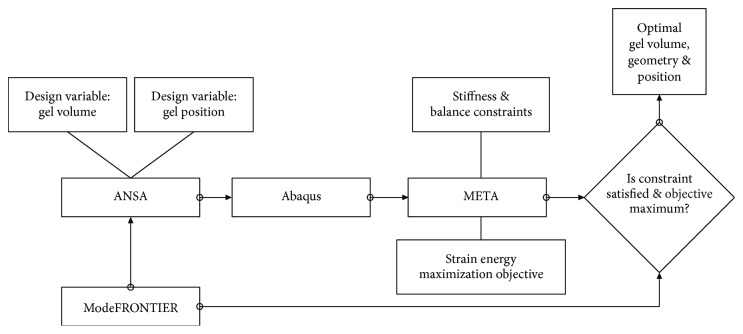
Logical diagram of the optimization setup. As shown in the logic diagram, both the influence of the position and volume of the gel pads were considered design parameters of the same loop and thus optimized in a single setup of the function/model.

Once the model was setup, it was subjected to a dynamic analysis in Abaqus/Explicit (Dassault Systèmes Simulia Corp., Providence, RI, USA). Following the initial simulation, the results were processed in META (by BETA CAE Systems S.A., Switzerland), as to determine the energy absorbed within the midsole as well as to calculate sole stiffness and displacement balance (proper support). Linking this workflow directly through modeFRONTIER facilitated the automated setup of all design variables. The parameters that control the shape and size of the gel inserts were created in the preprocessor ANSA (by BETA CAE Systems S.A., Switzerland), using mesh morphing methods. The logical diagram of the optimization process is demonstrated in Figure 4.

## 3. Results

As expected, the nonlinear composite (gel and EVA foam) midsole material exhibited a strain rate-dependent response, confirming recent experimental findings [22]. This confirms that only limited information can be obtained as to the system's shock absorbing capacity through quasistatic experiments or FE analyses.

The model revealed that the max von Mises stress, developing on the midsole, was in the range of 6.2 MPa (prior to optimization), observed during impact peak (around 0.09 ms of stance phase) on the lateral side of the foot, in between the calcaneus and the cuboid. This coincides well with the timing of the max plantar pressure (0.854 MPa), while stressing the significant force dissipation throughout the midsole structure.

**Figure 5:**
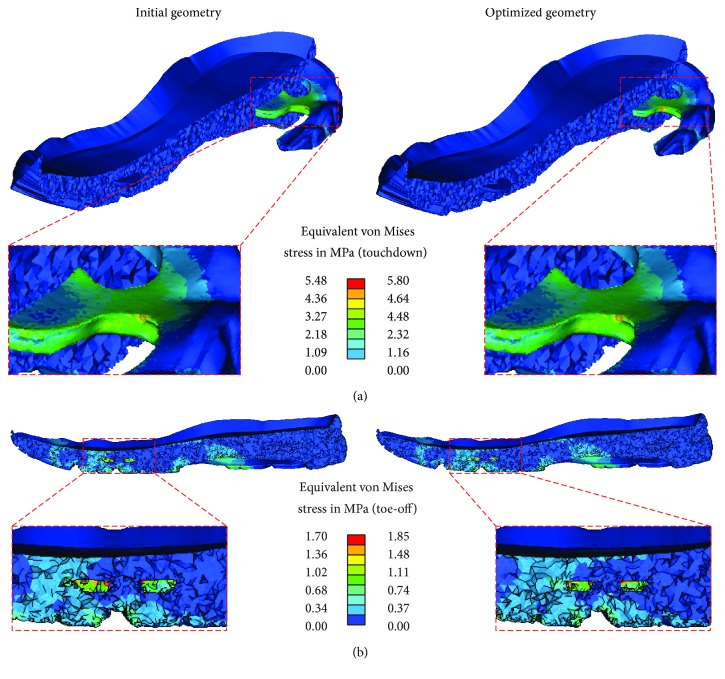
Varying stress distributions in the initial vs. optimized gel pad configuration during (a) touchdown and (b) toe-off.

Figure 5 highlights changes in the stress concentrations observed in both the gel pad and the EVA foam. Despite the similarities in the developing stress fields and the trends observed, transition of peak stress from gel in the heel pad region (Figure 5(a)) to the one placed under the central forefoot (Figure 5(b)), the developing stress patterns were clustered slightly differently while exhibiting max values up to 9% apart. The higher stress range in the optimized gel pad configuration indicates the higher strain energy absorbed in this scenario.

The evident localization of the in situ developing stress fields (Figure 5) suggests that the shock dissipating properties, although notable, could be significantly optimized as to provide increased cushioning. This was apparent when altering the position of the gel pads as well as during the modulation of their volume, since both led to different midsole responses. The max developing von Mises stress (within the midsole structure) could be altered by more than 40%, but these changes should only be considered with respect to the provided angular stability (a constraint, considered during optimization).

**Figure 6:**
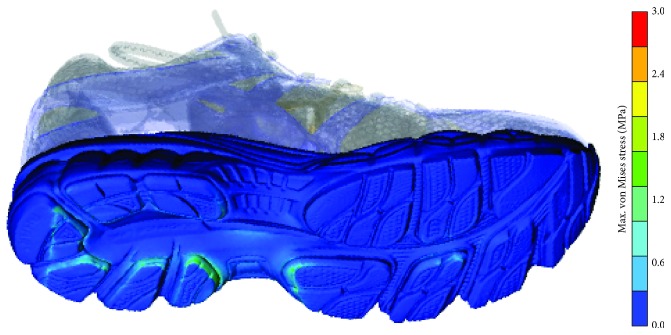
Equivalent von Mises stress distribution during an intermediate step of impact peak.

Characteristic stress fields, developing on the lower surface of the midsole (during impact peak), are illustrated in Figure 6, whereas the upper shoe structure is shown only to improve the 3D perception of the model.

Notably, several other regions of the midsole system exhibited stress accumulations, irrespectively of the gel placement and shape. This can be attributed, among others, to the isotropic nature of the implicated materials, stipulating that footwear design could further improve through the use of advanced and/or functionalized materials.

The pronation-specific placement of the secondary gel pad was vital to the sequential cushioning, provided in the rear-, mid-, and forefoot region of the shoe. The lesser magnitude of the ground reaction force transferred to the foot, in the latter two regions, indicated a smaller gel volume as sufficient to accommodate proper impact mitigation and stance support.

**Figure 7:**
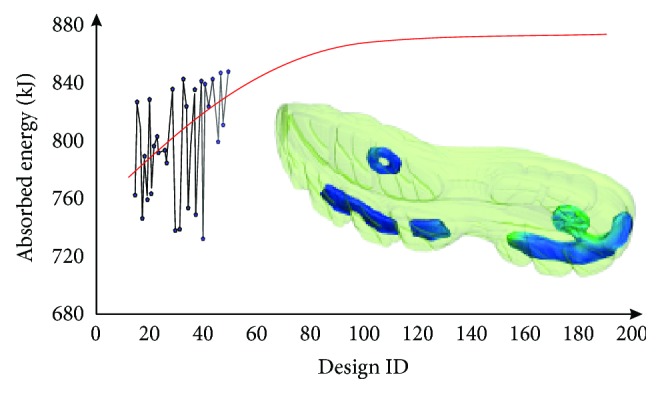
Convergence of optimization analysis towards the optimal solution.

The optimization function leads to the analysis of 200 gel pad variations (Figure 7), approximating a geometry and position that compared favorably to all prior examined scenarios.

The asymptomatic convergence of all examined geometries towards the optimum one indicates that further improvements are likely to only have an incremental impact on the midsole's absorption capacity, without disturbing the provided support.

**Figure 8:**
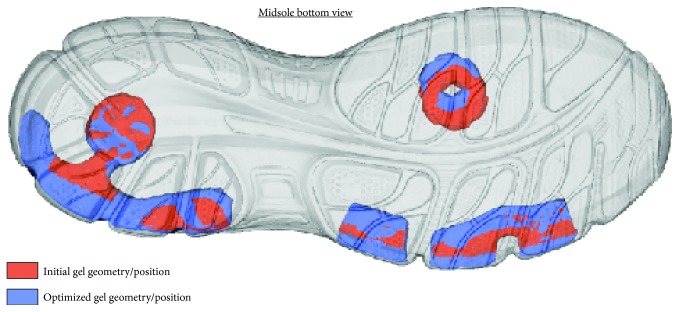
Initial and optimal gel pads (superimposed on the midsole structure).

The initial and optimized gel positions and volumes are superimposed, within the midsole structure, in Figure 8. It is noteworthy that the overall gel volume was varied by less than 10%, while the final geometry provided even better stability for the simulated gait pattern than the initial material allocation.

## 4. Discussion

Footwear and orthoses are called to fulfill multiple requirements [23], with cushioning and stability being among the most essential ones. Literature has repeatedly stressed the importance of several aspects, vital to shoe design and/or selection, such as anthropometric data [21], foot qualification [24], and gait depending traits [25].

The influence of shoe design on plantar pressure has been exhaustively investigated in vivo in the past [26, 27], although most studies conclude that predicting the effect of therapeutic footwear on an individual scale is limited by interpatient variability and environmental cues. As a result, the use of experimental approaches is restricted to evaluative purposes, rather than as a modality for preemptive design optimization.

Shariatmadari et al. [28] suggested FE modelling as a viable adjunct to heuristic methodologies, when addressing shape modification/optimization of footwear. In their study, a nonlinear synergetic response of midsole and insole was observed. This coincides well with the deformation mode of the midsole system examined here, as its stability was highly dependent on both the gel position and volume. Considering this as a constraint in the evolutionary computational approach applied in our study ensured that the optimized midsole geometry would not affect the angular position of the calcaneus, thus not biasing foot pronation of the athlete.

The mitigating properties of the midsole are evident when comparing results and plantar pressure measurements during other stance phases as well. A recent study of barefoot gait by Chen et al. [29] indicated the peak stress under the 3^rd^ metatarsal bone, amounted to 6.91 MPa during toe-off. In the shod running scenario, simulated here, the max plantar pressure during toe-off was less than 0.01 MPa. This can be attributed to the redirection of the developing loads to the midsole material, as the max stress within the shoe structure during these stages amounted to 2.1 MPa. It is noteworthy however that this stress relief is rather high, when compared to studies directly counter-examining shod to barefoot scenario [30], where variations are limited to 34%. This can be attributed to the load and boundary conditions of the study by Chen et al. [29], which varied significantly to the ones presented here.

Even though several other experimental studies report different max. and mean plantar pressure values [31], these inconsistencies are to be expected, due to intersubject variability and varying measurement techniques. Therefore, the important factor to consider is the recorded trend, rather than the measured value.

Shimoyama et al. [32] employed a similar FE-based methodology to the one presented here, formulated on equivalent design problem formulations. Next to the single-objective constraint optimization utilized in our case, their group also considered a multiobjective nonconstraint approach, to achieve faster conversion. Despite applying these evolutionary methods in a simplified design study, modulating the elastic modulus of the examined midsole system in three domains, some of their observations were sustained by the results presented here. This is also the case when considering the sensitivity to design parameters. In an effort to avoid violations of the stability constraint, our optimization function (similar to theirs [32]) proved less sensitive to design variables than expected. It would thus be preferable to focus on the constraint-handling method, e.g., a Pareto-based concept [33] examining the degree of violation, rather than a nonviolating criteria approach.

The low sensitivity of the optimization process to the design variables is among the limitations of this study. Even though the optimization function leads to an increase of about 8% in the strain energy (indicating a more effective force allocation of the impact between the foot and shoe), the rather small alteration in both gel pad volume and position is indicative of the functions inability to drive away for local optimums. This would suggest that an effective redesign of the midsole would require the subjection of multiple starting geometries/positions of the gel pads to the same methodology as to converge to the overall optimum.

Another aspect limiting the predictive capacity of the model lies in the evaluation of a single loading scenario, e.g., heel strike pattern. Future work should consider variations in strike pattern and pronation, while also adjusting these with respect to the optimized results, as running is widely considered a self-optimizing activity [34] and thus a subject's gait expected to adjust even to these minor changes.

The inclusion of a foot model will also be considered in the future. Despite the fact that the implementation of such foot-shoe interactions [35, 36] arrives at similar conclusions as shown here, considering tissue properties, would provide refined insight to tissue-specific loading. This could be vital for patients in need of prescribed therapeutic footwear, i.e., patients suffering from diabetes and peripheral neuropathy [37], as ground reaction forces have been associated with lower extremity skin breakdown.

## 5. Conclusions

This investigation introduced a dynamic FE model of a running shoe, considering time-varying plantar pressure distributions and boundary conditions. The model was used to suggest improvements of material allocation within the midsole system, with energy absorption as optimality criterion and stability as a constraint. The results of the study revealed a nonlinear relationship of midsole material allocation to the optimization parameters, exhibiting however a profound effect on both cushioning and stability.

The results confirmed the hypothesis of the study, suggesting that midsole design could be significantly improved through the use of biorealistic FE modelling techniques and proper optimization functions, providing a new platform for the conceptual redesign and/or optimization of modern footwear.

## Data Availability

All data used in the present work is available, and given consent of all involved authors is provided for the access to any requested information, data, or raw files.
